# The diagnosis of male infertility: an analysis of the evidence to support the development of global WHO guidance—challenges and future research opportunities

**DOI:** 10.1093/humupd/dmx021

**Published:** 2017-07-19

**Authors:** Christopher L R Barratt, Lars Björndahl, Christopher J De Jonge, Dolores J Lamb, Francisco Osorio Martini, Robert McLachlan, Robert D Oates, Sheryl van der Poel, Bianca St John, Mark Sigman, Rebecca Sokol, Herman Tournaye

**Affiliations:** 1 Department of Reproductive and Developmental Biology, Medical School, Ninewells Hospital, University of Dundee, Dundee, DD1 9SY, Scotland; 2 Karolinska University Hospital and Karolinska Institutet, Stockholm, Sweden; 3 University of Minnesota, Minneapolis, MN, USA; 4 Baylor College of Medicine, Houston, TX, USA; 5 Clinica Alemana de Santiago, Santiago, Chile; 6 Hudson Institute of Medical Research, Clayton, Australia; 7 Boston University School of Medicine and Boston Medical Center, Boston, MA, USA; 8 Department of Reproductive Health and Research, Human Reproduction Programme, (HRP, The UNDP/UNFPA/UNICEF/WHO/World Bank Special Programme of Research, Development and Research Training in Human Reproduction), WHO, Geneva, Switzerland; 9 Population Council, New York, NY, USA; 10 Hudson Institute of Medical Research, Clayton, Australia; 11 Warren Alpert Medical School of Brown University, RI, USA; 12 Department of Obstetrics and Gynaecology and Medicine, Keck School of Medicine, University of Southern California, CA, USA; 13 Centre for Reproductive Medicine, Vrije Universiteit Brussel, Brussels, Belgium

**Keywords:** male infertility, spermatozoa, genetics, Y deletions, cystic fibrosis transmembrane conductance regulator, semen analysis, varicocele, evidence-based guideline, cancer

## Abstract

**BACKGROUND:**

Herein, we describe the consensus guideline methodology, summarize the evidence-based recommendations we provided to the World Health Organization (WHO) for their consideration in the development of global guidance and present a narrative review of the diagnosis of male infertility as related to the eight prioritized (problem or population (P), intervention (I), comparison (C) and outcome(s) (O) (PICO)) questions. Additionally, we discuss the challenges and research gaps identified during the synthesis of this evidence.

**OBJECTIVE AND RATIONALE:**

The aim of this paper is to present an evidence-based approach for the diagnosis of male infertility as related to the eight prioritized PICO questions.

**SEARCH METHODS:**

Collating the evidence to support providing recommendations involved a collaborative process as developed by WHO, namely: identification of priority questions and critical outcomes; retrieval of up-to-date evidence and existing guidelines; assessment and synthesis of the evidence; and the formulation of draft recommendations to be used for reaching consensus with a wide range of global stakeholders. For each draft recommendation the quality of the supporting evidence was then graded and assessed for consideration during a WHO consensus.

**OUTCOMES:**

Evidence was synthesized and recommendations were drafted to address the diagnosis of male infertility specifically encompassing the following: What is the prevalence of male infertility and what proportion of infertility is attributable to the male? Is it necessary for all infertile men to undergo a thorough evaluation? What is the clinical (ART/non ART) value of traditional semen parameters? What key male lifestyle factors impact on fertility (focusing on obesity, heat and tobacco smoking)? Do supplementary oral antioxidants or herbal therapies significantly influence fertility outcomes for infertile men? What are the evidence-based criteria for genetic screening of infertile men? How does a history of neoplasia and related treatments in the male impact on (his and his partner's) reproductive health and fertility options? And lastly, what is the impact of varicocele on male fertility and does correction of varicocele improve semen parameters and/or fertility?

**WIDER IMPLICATIONS:**

This evidence synthesis analysis has been conducted in a manner to be considered for global applicability for the diagnosis of male infertility.

## Introduction

**Figure 1:**
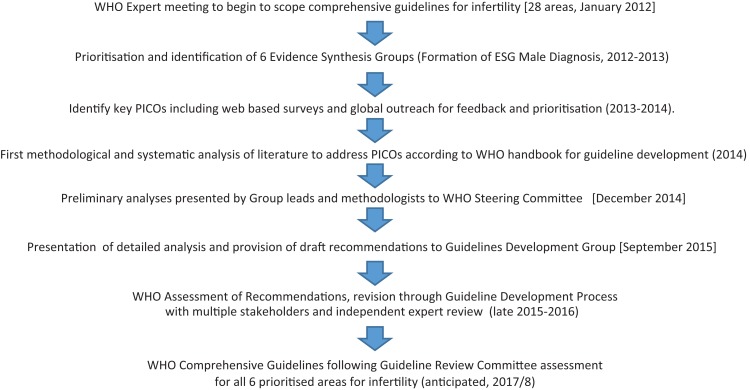
Outline flowchart of WHO methodology for ESG Male Diagnosis. Flowchart outlining the WHO process for obtaining the evidence, and formulating and presenting recommendations for male infertility (Diagnosis). This includes stages and methods for synthesis of evidence according to WHO process. Dates in square bracket reflect specific meetings at WHO in Geneva. PICO: problem or population (P), intervention (I), comparison (C) and outcome(s) (O). WHO, World Health Organization; ESG, Evidence Synthesis Group.

In 2012, the World Health Organization (WHO) held a meeting of experts to scope the field of fertility care in order to develop comprehensive guidelines on infertility. Six Evidence Synthesis Groups (ESG) were established. One group was the WHO ESG on Male Infertility: Diagnosis. Following the initial meeting, key PICO (problem or population (P), intervention (I), comparison (C) and outcome(s) (O)) questions were developed and agreed upon. This included working through the WHO GDG (Guideline Development Group) Committee, as well as through web-based surveys, and through outreach to developing country scholars taking an on-line Geneva Foundation for Medical Education and Research- American Society for Reproductive Medicine- (GFMER-ASRM) WHO evidence-based infertility course. For each PICO question, a systematic analysis of the literature was performed according to the ‘WHO handbook for guideline development’ (WHO, 2014). A preliminary analysis of the data was presented to the WHO/GDG Steering Committee Working Experts Consultation in December 2014 during which modifications were made to various components of a few of the PICO questions, and additional PICOs were also identified. A comprehensive document including draft recommendations was presented to the WHO/GDG Steering Committee Meeting for Guidelines and Nomenclatures in September 2015 (Fig. 1). This manuscript provides a narrative review of the evidence synthesized by the ESG that helped to generate the recommendations (Table I), provides an update of the evidence as recommended through expert review, defines some of the challenges in addressing these questions and discusses current research gaps. It concludes by presenting future research opportunities and outlines how these may be realized.

**Table I:** Male Factor Infertility Diagnosis: Summary Recommendations.

Clinical questions	RECOMMENDATIONS through assessment of developed PICO question and associated evidence analysis	Strength of the evidence
1. What is the prevalence of male infertility and what proportion of infertility is attributable to the male?	It is not possible to determine an unbiased prevalence of male infertility in the general population.	Very low
2. Is it necessary for all infertile men to undergo a thorough evaluation?	The initial evaluation for male factor infertility should include a PE performed by an examiner with appropriate training and expertise, a reproductive history and at least one properly performed (high quality) semen analyses. A full evaluation by a urologist or other specialist in male reproduction should be done if the initial screening evaluation demonstrates an abnormal PE, an abnormal male reproductive or sexual history, or an abnormal semen analysis is found. Further evaluation of the male partner should also be considered in couples with unexplained infertility and in couples in whom there is a treated female factor and persistent infertility	Moderate
3. What is the clinical (ART/non ART) value of traditional semen parameters?	Assessment of a combination of several ejaculate parameters is a better predictor of fertility success than a single parameter	High
	Analysis of a single ejaculate is sufficient to determine the most appropriate investigation and treatment pathway although semen analysis could be repeated if one or more abnormalities is found	High
4. What key male lifestyle factors impact on fertility?	Evidence supports a detrimental effect of obesity on many aspects of health; evidence is conflicting about a potential effect on reproductive function. Males presenting for fertility evaluation should be counseled about weight-loss strategies when the BMI and waist circumference data demonstrate obesity and especially morbid obesity.	Moderate
	There is insufficient evidence to conclude that exposure to heat, be it occupational or as a result of clothing or body position, affect semen quality and/or male fertility	Very low
	There is some evidence to suggest a negative effect of cigarette (tobacco) smoking on semen quality but not all studies report this. However, as smoking has an adverse effect on general health and wellbeing it is recommended that men trying for a pregnancy should abstain from smoking	Moderate
5. Do supplementary oral antioxidants or herbal therapies significantly influence fertility outcomes for infertile men?	There are insufficient data to recommend the use of supplemental antioxidant therapies for the treatment of men with abnormal semen parameters and/or male infertility	Low
	There are insufficient data to recommend the use of herbal therapies for the treatment of men with abnormal semen parameters and/or male infertility	Very low
6. What are the evidence-based criteria for genetic screening of infertile men?	Karyotype testing should be performed on all males with severe oligozoospermia (<5×10^6^/ml) or NOA prior to any therapeutic procedure	High
	YCMD testing should be performed on all males with severe oligozoospermia prior to a therapeutic procedure or NOA prior to any therapeutic procedure	High
	Appropriate *CFTR* mutation analysis should be offered to all males with CBAVD or CF	High
7. How does a history of neoplasia and related treatments in the male impact (his and his partner's) reproductive health and fertility options?	Every male cancer patient should be provided with information about the impact of his cancer treatment on spermatogenesis and the option of sperm banking	Moderate
	Patients should be advised to use contraception if they do not wish to procreate even after prolonged periods of azoospermia following radiotherapy, as recovery is possible	Low
	Male cancer patients should be informed that pregnancy outcomes in partners of male cancer survivors are good but a slightly higher risk of congenital anomalies in their offspring cannot be excluded	Low
8. What is the impact of varicocele on male fertility and does correction of varicocele improve semen parameters and/or fertility?	Good Practice Point: Treatment of a clinically palpable varicocele may be offered to the male partner of an infertile couple when there is evidence of abnormal semen parameters and minimal/no identified female factor, including consideration of age and ovarian reserve	Very low
	Good Practice Point: IVF with or without ICSI may be considered the primary treatment option when such treatment is required to treat a female factor, regardless of the presence of varicocele and abnormal semen parameters	Very low
	Good Practice Point: The treating physician's experience and expertise, including evaluation of both partners, together with the options available, should determine the approach to varicocele treatment	Very low

PICO, problem or population (P), intervention (I), comparison (C) and outcome(s) (O); CBAVD, Congenital Bilateral Absence of the Vas Deferens; PE, physical examination; YCMD, Y chromosome microdeletion; NOA, non-obstructive azoospermia.

## Methods

### The key PICO questions

The following eight topics were pre-determined and identified by WHO, and were later formulated into PICO questions for systematic analysis, as follows:

- What is the prevalence of male infertility and what proportion of infertility in the couple is attributable to the male?
- Is it necessary for all infertile men to undergo a thorough evaluation?
- What is the clinical (ART/non ART) value of traditional semen parameters?
- What key male lifestyle factors impact on fertility (focusing on obesity, heat and tobacco smoking)?
- Do supplementary oral antioxidants or herbal therapies significantly influence fertility outcomes for infertile men?
- What are the evidence-based criteria for genetic screening of infertile men?
- How does a history of neoplasia and related treatments in the male impact on (his and his partner's) reproductive health and fertility options?
- What is the impact of varicocele on male fertility and does correction of varicocele improve semen parameters and/or fertility?

### Outline evidence synthesis methodology

The methodology used to support the provision of the recommendations was outlined by the WHO (2014) namely: identification of priority questions and critical outcomes; retrieval of up-to-date evidence and existing guidelines; assessment and synthesis of the evidence; and formulation of draft recommendations to be used for reaching a consensus with a wide range of global stakeholders. For each recommendation the quality of the supporting evidence would then be graded (very low, low, moderate and high) for consideration for the consensus. For example, a rating of high quality of evidence means that further research is very unlikely to change our confidence in the estimate of the effect. Conversely a rating of very low quality of evidence means that any effect is very uncertain.

We qualified the strength of our recommendations (as strong or weak) based upon consideration of the quality of the evidence. These recommendations were then later assessed through the WHO guideline development processes that are based upon other factors including values and preferences of stakeholders, the magnitude of effect, the balance of benefits versus harm, resource used and the feasibility of implementation.

Overall, wherever possible, original literature, and data from recently published systematic and Cochrane reviews were used. However, due to the diagnostic nature of the questions, a significant amount of the literature addressing them does not lend itself to high evidence level RCTs. Therefore, in addition, key guidelines and professional committee opinions were examined, in order to assist in developing a broader and more comprehensive evidence base, as well as in the construction of recommendations, and in particular: ASRM—Diagnostic evaluation of the infertile male: a committee opinion (2015) (ASRM, 2015a); American Urological Association [AUA] Best Practice Statements (updated 2010 and 2011); EAU Guidelines on Male Infertility (Jungwirth *et al.*, 2015); and the Society for Male Reproduction and Urology (2014) Report on varicocele and infertility.

Data were presented in summary form and descriptively, in tables or narratively in the evidence reviews for each PICO question. Where appropriate, meta-analyses were conducted. The GRADE framework was applied to the body of evidence for each outcome within each PICO. The WHO then used the worksheets to summarize the volume and quality of the evidence supporting the recommendations as well as to outline the values, preferences and judgements made about the strength of recommendations. The uniqueness of the WHO process was that these balanced worksheets were also to be used to note considerations especially for low- and middle-income countries or settings, and to be able to record the reasons for changes made to the default strength of the recommendations.

The principles or best practice guidance need to be consensus-based and are also intended to underscore the importance of respect for reproductive rights and dignity as recipients of care, and the need to maintain high ethical and safety standards in clinical practice. These principles, in addition to the strategies for implementation, monitoring and evaluation, are expected to guide end-users in the process of adapting and implementing any recommendation provided by the WHO to consider for a range of global contexts and settings.

The evidence based and detailed analysis, with GRADE tables where possible for each of the prioritized PICO questions, were commissioned by and provided by the first author to the WHO in support of their guideline processes. A WHO assessment of our evidence-based outcomes was then undertaken with many stakeholders who evaluate other factors including values and preferences of stakeholders, the magnitude of effect, and the balance of benefits versus harms, resource use and the feasibility of implementation to better assure global applicability. As required by WHO, following these outcomes, additional independent expert review would be conducted (2016 and early 2017). Once completed, the WHO will be publishing their expert and stakeholder consensus-driven guidelines together with the detailed evidence base (evidence tables, detailed search strategies, balanced worksheets etc.) and related products (Fig. 1).

The present manuscript provides a narrative of the evidence. It particularly focuses on areas where evidence is controversial, of poorer quality and more challenging to obtain. It discusses what is missing from the analysis and critically provides a discussion of potential research gaps. It is not the purpose of this manuscript to reproduce the original documents submitted to the WHO. As it was necessary to undergo a global prioritization method to identify answerable PICO questions, there inevitably are a number of questions in the diagnosis of male infertility not addressed by the WHO ESG and thus absent from this manuscript. For example, the effect of paternal age, alcohol and environment on male fertility (those interested can consult for example: Age—Eisenberg & Meldrum, 2017; Nybo-Andersen and Urjoj, 2017; Johnson *et al.*, 2015; Ramasamy *et al.*, 2015; Alcohol—Karmon *et al.*, 2017; Jensen *et al.*, 2014; Oil and natural gas extraction—Balise *et al.*, 2016; Bisphenol A—Mínguez-Alarcón *et al.* 2016; Outdoor air pollution—Lafuente *et al.*, 2016).

## Summary of outcomes and narrative review as related to the eight prioritized PICO questions

The specific recommendations formulated for the presentation to the WHO on the diagnosis of male infertility are included in the text along with an assessment of the quality of the supporting evidence (Table I) and strengths of recommendations based upon our evidence synthesis. The final recommendations will only result following an independent expert review of our work and review by stakeholder societies, following assessment through the Guidelines Review Committee of WHO.

### What is the prevalence of male infertility and what proportion of infertility is attributable to the male?

This is a simple and fundamental question. It is critical to know the prevalence of a disease in order to provide resources, estimate impact, make effective health economic arguments, present rational research questions and manage patients. Investigators studying other diseases often have the incidence of the disease well established in a variety of different populations. However, for male infertility this remarkably simple question is surprisingly very difficult to answer.

The most recent publication presents a population prevalence estimate of infertility amongst 15 162 men and women in the UK (Datta *et al.*, 2016). This was a cross-sectional survey asking if the participants had ever had a time, lasting 12 months or longer, when they and their partner were trying for a pregnancy but it did not happen. One in eight women (12.5%, 95% CI 11.7–13.1) and one in ten men (10.1%, 95% CI 9.2–11.1) answered yes to this question and thus had experience of infertility. This type of study needs repeating in a number of different geographical regions.

Addressing the prevalence of male infertility is a challenging one. For example, a difficulty arises from a lack of continuity in the definitions of infertility. Generally, infertility is defined as failure of a ‘couple’ to become pregnant despite 12 or more months of unprotected intercourse. However, some studies such as Hull *et al.* (1985) include in their definition couples who become pregnant but miscarry. Other studies such as Anderson *et al.* (2009) and Gurunath *et al.* (2011) include those who seek medical advice in order to be able to make a partner pregnant. There is no current method to capture men as individuals or in same-sex relationships, who may desire a biological child through ART, and who may be found to be infertile. Furthermore, there is also generally a lack of differentiation between primary and secondary infertility in the heterosexual male, and the relative rates of primary and secondary infertility vary significantly between studies, especially when comparing clinic-based and population-based studies (see Malekshah *et al.*, 2011 versus Klemetti *et al.*, 2010).

The varying definitions used for male infertility and the fact that men are not always evaluated (Pastuszak *et al.*, 2016) can result in misleading study conclusions. Mehta *et al.* (2016) recently documented many of these obstacles when trying to obtain an accurate assessment of the prevalence of male infertility in the USA. Consequently, it is not surprising that there are currently no rigorous systematic reviews or meta-analyses on the epidemiology of male infertility. Agarwal *et al.* have attempted to pursue this type of review (Agarwal *et al.*, 2015) but due to a paucity of high-quality comparable studies they were unable to make robust conclusions. There are significant variations in the variables assessed between studies, including, but not limited to the age of the participants, the participants themselves (individual males, females or couples), the method of data collection and the outcomes measured. These caveats create inconsistencies in the study results, and consequently, studies of male infertility can generally be divided into two categories: those that seek to determine the prevalence or incidence of the experience of infertility amongst men, or those which focus on the proportion of total infertility that is attributable to the male factor. For example, it is insufficient to simply ask men if they experienced infertility because this information does not give a true representation of male factor infertility (as their partner could be the cause of the infertility). On the other hand, clinical studies of diagnosed male factor infertility itself often suffer from small sample size (Geelhoed *et al.*, 2002) or a biased population—those that consult—which could skew the data. Not all couples experiencing infertility choose to consult a physician and, of couples that do consult, not all will have experienced greater than 12 months of infertility. For example, Louis *et al.* (2013) reported a higher prevalence of infertility than Anderson *et al.* (2009) using the same study population, because the latter's definition of infertility was restricted to men who had gone for a consultation. Similarly, van Roode *et al.* (2015) reported a higher prevalence of infertility amongst 38-year-old men (18.3% compared to 14.4%) when they expanded their definition of infertility to include those who had sought medical help to generate a pregnancy. The selection of subject populations is often inherently biased. And in the case of assessing male infertility, for example, studies performed on specific populations, such as military recruits, may not reflect the general population. Large-scale studies of the prevalence of infertility generally focus on women's experience of infertility as reported in demographic surveys which are based upon contraceptive usage (e.g. Gurunath *et al.*, 2011, Mascarenhas *et al.*, 2012) and few large-scale studies are able to gather data on men. Van Roode *et al.* (2015) reported a large difference in the diagnosis of fertility problems when asking women in a survey compared to asking men.

Current studies of male infertility often employ cross-sectional population study designs (e.g. Datta *et al.*, 2016 above), or are observational studies of those men who present to infertility clinics. Only one prospective birth cohort study was identified (van Roode *et al.*, 2015). A further limitation of the available literature is that some studies are relatively old, such as Hull *et al.* (1985) and Thonneau *et al.* (1991); studies which have not been updated in a quarter of a century. There may also be a geographical variation in the incidence of male infertility—one study in France suggested that male factor alone accounted for 20% of total infertility (Thonneau *et al.*, 1991) whilst a study in Western Siberia put the figure at 6.4% (Philippov *et al.*, 1998). It is unclear whether these represent true geographical variations or simply differences in methodology.

Suffice it to say that, based on current evidence, few reliable conclusions can be drawn about the epidemiology of male infertility. Several studies have suggested that male factor infertility is the single most common diagnosis among heterosexual couples who struggle to become pregnant but definitions and diagnosis of male factor vary and several other studies report that female factor infertility is more prevalent. Nevertheless, all of these studies highlight the need for further research.

**Figure 2:**
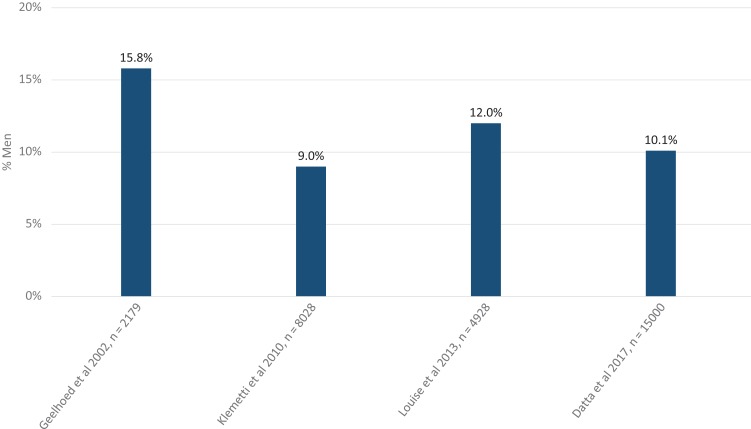
Prevalence of male infertility. Prevalence of male infertility in surveys of general populations. Male infertility was generally defined as men reporting experience of infertility (generally >12 months in duration).

One simplistic and frequently used approach to assess male infertility has been to examine semen parameters in men of the general population and determine the frequency of semen abnormalities against standard ranges (Cooper *et al.*, 2010, Virtanen *et al.*, 2017). A plethora of studies have done this and also used this information in an attempt to address changes in semen quality over time (Virtanen *et al.*, 2017). The advantage of these studies is that they can provide comparable data but only if first the populations are well characterized and second the laboratory methods used to determine semen quality are robust and consistent across study sites (cf. Björndahl *et al.*, 2016). However, the primary disadvantage of this approach is that semen parameters alone are not equivalent to defining infertility/fertility (MacLeod, 1950; Guzick *et al.*, 2001; Cooper *et al.*, 2010). As such, the focus of the current analysis was on the proportion of heterosexual men who experience a delay (extended time) in inducing a pregnancy (Fig. 2).

In summary, we strongly recommend, owing to the very low quality of evidence, that it is not currently possible to determine an unbiased prevalence of male infertility within the global, regional or national populations, including neglected individual populations. Additionally, it is not currently possible to determine what proportion of infertility in heterosexual couples is attributable to the male partner (Table I).

A number of topics were identified for future research. There is a need for large population-based studies to determine the prevalence of male infertility in the general population of males whether in a relationship or not. Ideally, large population-based cohort studies conducted in a number of different geographical regions must be carried out with consistent definitions of infertility and comparable clinical study designs.

### Is it necessary for all infertile men to undergo a thorough evaluation?

Medical conditions in the male may be causative of the infertility (such as hypogonadotropic, hypogonadism or bilateral cryptorchidism) or associated with the infertility (testis tumour in male with normal semen analysis). The rationale for evaluating the male and the extent of that evaluation depends on the goals of the evaluation. Several medical best practice statements (AUA, 2011; ASRM, 2015a) suggest that the goals of the evaluation of the male are to identify: conditions that can be corrected; conditions that are irreversible for which ART will be needed using the male partner's sperm; irreversible conditions for which the male partner's sperm will not be available or appropriate and may require consideration of donor sperm or adoption; serious medical conditions that may be causing or present with male infertility and that could affect the health of the male and require medical treatment; and genetic causes of male infertility that could affect the success of treatment or the health of offspring if ART is utilized.

Evaluations of populations of infertile men have identified patients in each of these categories emphasizing the need for evaluation of the male (Nieschlag and Behre, 2001; Tournaye *et al.*, 2016; Olesen *et al.*, 2017; Punab *et al.*, 2017, Pastuszak *et al.*, 2016). An initial evaluation of the male consists of three primary components: history, physical examination (PE) and semen analyses. There is general agreement about the importance of obtaining a reproductive history (including a sexual history) and semen analyses. The ASRM (ASRM, 2015a) suggest that the reproductive history should include: coital frequency and timing; duration of infertility and previous fertility; childhood illnesses and developmental history; systemic medical illnesses (such as diabetes mellitus and upper respiratory diseases); previous surgery; medications and allergies; sexual history (including sexually transmitted infections); and exposures to gonadotoxins (including environmental and chemical toxins and heat). A discussion point, however, is the need, timing of investigations, and indications for PE; and it is in this area where the current guidance appears to be inconsistent. Practice statements by the AUA and ASRM recommend an initial evaluation of all males of infertile couples that consists of a detailed reproductive history and semen analyses. For example, the ASRM states ‘At a minimum, the initial screening evaluation of the male partner of an infertile couple should include a reproductive history and analysis of at least one semen sample’ (ASRM, 2015a). The ASRM then recommends that those men with risk factors in their reproductive history or abnormal semen parameters should be referred to a male reproductive specialist for a more thorough evaluation that includes a PE. Notably, both organizations (ASRM and AUA) recommend consideration of a full male evaluation (including PE) in those couples with unexplained infertility or those that remain infertile after correction of female factors. The European Association of Urology (EAU) states ‘A medical history and PE are standard assessments in all men’ (Jungwirth *et al.*, 2015). Others have suggested a full evaluation of all men in infertile relationships (Honig *et al.*, 1994; Kolettis and Sabanegh, 2001; Olesen *et al.*, 2017; Punab *et al.*, 2017), which includes a PE.

The question is: Which diagnostic strategy is optimal? A significant number of identified male factors are associated with abnormalities found by semen analysis. However, the aetiology remains to be robustly quantified, especially in light of causes of infertility such as birth defects, acquired forms of infertility, infection, inability to have an erection or ejaculation, various syndromes as well as metabolic and endocrine disorders. Sexual dysfunction may be associated with normal semen parameters but can be identified through a sexual history. Additionally, there are a number of genetic syndromes that predispose or cause male infertility some of which are associated with abnormal semen parameters. An initial evaluation of the male consisting of a reproductive history, a simple PE and semen analysis would potentially identify the majority of these cases, however, these evaluations must also be sensitive to different cultural practices and different regional aetiologies, e.g. HIV, genital tuberculosis (TB) in TB endemic areas, lifestyle, environmental and occupational hazards. Significant medical conditions have been reported in 1.1–6% of men presenting for infertility evaluations and a number of these men have abnormal semen parameters. Importantly, however, there are limited data on the incidence of significant medical conditions that predispose men to infertility. One older data series reported that 0.16% of men had significant medical conditions but normal semen parameters indicating that conditions will be missed by limiting a male assessment to a reproductive history and semen analysis (Honig *et al.*, 1994).

Consequently, there are several approaches that can be utilized consisting of the following possibilities. One approach is that all sub-fertile men should have an initial evaluation with history, PE by an examiner with appropriate training and expertise, and a semen analysis. Importantly, this will pick up conditions missed by excluding a PE and is consistent with the EAU guidelines (Jungwirth *et al.*, 2015). Alternatively, initial evaluation consists of detailed reproductive history and semen analyses; in this scenario, only if either is abnormal does the male undergo a more thorough history and a PE. However, men with significant medical conditions will be missed by this approach. On balance, there are, on a global scale, a number of advantages and few disadvantages to include a PE performed by an examiner with appropriate training and expertise as part of this initial evaluation. For example, in low- to middle-income countries where visits to infertility health professionals will be more restricted due to factors such as geography and costs, it is less likely that a man will return to the clinic even if the results of a semen analysis are abnormal.

Interestingly, both ASRM and EAU ASRM do not recommend endocrine testing as a primary first line investigation. For example, the ASRM (2015a) suggest endocrine testing in men with abnormal semen parameters (particularly when the sperm concentration I < 10 million/ml), impaired sexual function or clinical findings that suggest a specific endocrinopathy. And, as ASRM state, some experts think that all infertile men merit an endocrine evaluation (Ventimiglia *et al.*, 2016; Olesen *et al.*, 2017). What is important is that the key recommendations are verified in different populations to establish how robust they are, and if required such recommendations are amended. Interestingly, Ventimiglia *et al.* have recently examined the ASRM indications for endocrine assessments in a cross-sectional study of 1056 infertile men to predict hypogonadism. Using the same database, the authors developed a logistic regression-based nomogram including testis volume measured during the physical exam, BMI and azoospermia to predict total testosterone levels of <3 ng/dl. Although, their nomogram had a higher predictive accuracy (68%) than ASRM's guidelines (58%), they concluded, based on their statistical analyses, that their nomogram also was not reliable enough to predict hypogonadism. These examples emphasize the importance of validating recommendations in a variety of populations.

**Figure 3:**
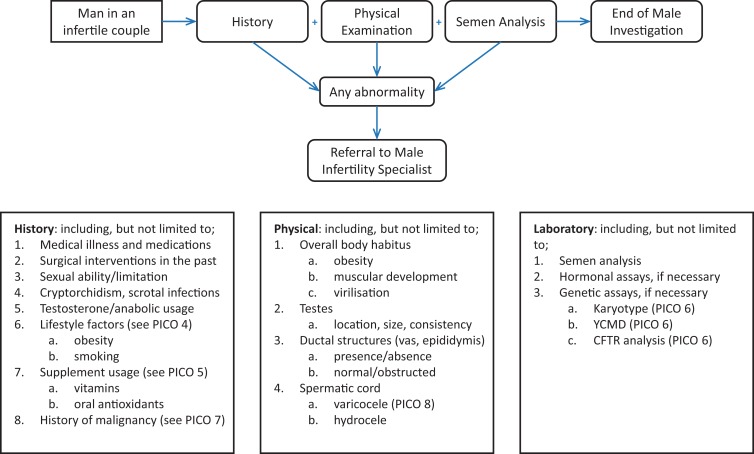
Flowchart summary of algorithm for diagnosis of male infertility. As detailed in section PICO 2 (Is it necessary for all infertile men to undergo a thorough evaluation?) the first line investigations should include Physical Examination, History and Semen Analysis. Abnormalities in these lead to further investigations. YCMD, Y chromosome microdeletion; CFTR, CF transmembrane conductance regulator.

In summary, we strongly recommend based on a moderate quality of evidence that:

- The initial evaluation for male factor infertility should include a PE performed by an examiner with appropriate training and expertise, a reproductive history, and at least one properly performed (high quality) semen analyses.
- A full evaluation by an urologist or other specialist in male reproduction should be carried out if the initial screening evaluation demonstrates an abnormal PE, an abnormal male reproductive or sexual history, or an abnormal semen analysis is found.
- Further evaluation of the male partner should also be considered in couples with unexplained infertility and in couples in whom there is a treated female factor and persistent infertility (Fig. 3, Table I).

There are significant areas for future research. For example, what constitutes the most appropriate minimal PE and does this provide significant additional information for male health in general, does performing a PE improve male engagement in diagnosis and treatment, what is the cost-effectiveness of doing a PE on all men attending an infertility clinic instead of doing a PE only after abnormal findings in semen analysis and/or an abnormal reproductive and sexual history, can a globally validated questionnaire/topic list for reproductive and sexual history be used (encompassing low-income settings) to identify individuals at risk for male infertility, and Is the outcome better with earlier diagnosis and is treatment less expensive with earlier diagnosis?

### What is the clinical (ART/non ART) value of traditional semen parameters?

Specifically, we posed two clinical questions. This first question was whether the predictive value of semen analysis for reproductive outcome is better using a combination of several parameters compared to a single semen analysis parameter, and the second was whether an evaluation of a single ejaculate versus two ejaculates is sufficient for referral to infertility investigation and treatment.

A fundamental challenge in the analysis of the literature is the quality of the laboratory testing which is often performed using sub-optimal methods (Keel, 2004; Björndahl *et al.*, 2016). This is illustrated by data presented from three recent national quality control programmes in Belgium Germany and Italy (Mallidis *et al.*, 2012; Filimberti *et al.*, 2013; Punjabi *et al.*, 2016) documenting that, in general, a number of laboratories do not adhere to WHO methods for semen analysis. Lack of adherence to recommended and appropriately standardized methods significantly undermines the potential diagnostic value. Effective strategies to address this issue remain a subject of significant debate (e.g. Carrell and De Jonge, 2016).

The analysis of studies to address this PICO question was limited to those published after 1 January 2000, based on two key assumptions. First, there has been a very slow increase in compliance with WHO recommendations for semen analysis. The fourth edition was published in 1999 and few publications before that bear evidence of significant and clear compliance with the recommended techniques (see Tomlinson *et al.*, 1999). Second, evaluation of the prognostic value of semen analysis is dependent on the quality of the clinical interventions available. It is, therefore, likely that ‘historic’ data for ART success are not relevant for treatments available in recent years as the ART techniques have significantly improved (Wade *et al.*, 2015).

Another major challenge which has been recognized for over 70 years is the substantial overlap in the distribution of semen analysis results in fertile men and those from men in infertile couples (MacLeod, 1950; Guzick *et al.*, 2001; Cooper *et al.*, 2010). This means that comparing a patient's semen parameters to the distribution of results for fertile men is not in itself sufficient to determine whether or not the patient is fertile or infertile (Björndahl, 2011). Additionally, semen analysis is only part of the investigation of the man and a number of other attributes contribute to his fertility potential.

With regard to the first question namely, Is the predictive value of semen analysis for reproductive outcome better using a combination of several parameters compared to a single semen analysis parameter? Studies were regarded as eligible if they presented primary data on predictive values [or odds ratios (OR)] concerning multiple or single analysis parameters. Four studies provided data on multiple assessments of parameters (Zinaman *et al.*, 2000; Guzick *et al.*, 2001; Jedrzejczak *et al.*, 2008; van der Steeg *et al.*, 2011, total *n* = 5022) and 10 studies focussed on single ejaculate parameters as predictors of fertility. Studies identifying a single semen parameter with significant predictive power for fertility are relatively common but the discriminatory power is very often low e.g. expressed as receiver operating characteristic curve/area under the curve (ROC–AUC) close to 0.500 or OR including 1.00 in the 95% CI. In contrast, studies that have investigated multiple parameters, which are more reflective of testicular production/function and maturation, for example Guzick *et al.* (2001) and Jedrzejczak *et al.* (2008), present OR or predictive values, that are comparable to diagnostic laboratory tools in other areas of modern clinical medicine (Boyd, 2010). As such the conclusion of this analysis was that examination of multiple parameters was more predictive.

In summary, we strongly recommend based on a high quality of evidence that the assessment of a combination of several ejaculate parameters is a better predictor of fertility success than a single parameter (Table I).

The second fundamental question is commonly posed but rarely answered: Is an evaluation of a single ejaculate versus two ejaculates sufficient for referral to infertility investigation and treatment? This was considered important globally, due to the low level of male engagement to address reproductive health issues in many settings. As above, the analysis was limited to studies published after 1 January 2000 primarily due to the presumed advances in quality in laboratory andrology. In this analysis, studies were only eligible if they provided appropriate information to ascertain the reliability of the data obtained from semen analysis, and they presented primary data concerning the usefulness of repeat analyses [e.g. intraclass correlation coefficient (ICC), where an ICC close to 1.00 indicates high reliability between a pair of assessments].

Only five studies (Francavilla *et al.*, 2007; Stokes-Riner *et al.*, 2007; Mishail *et al.*, 2009; Leushuis *et al.*, 2010; Christman *et al.*, 2013 total *n* = 6482) provided information that could be used to support or reject a recommendation concerning analysis of a single versus two ejaculates (see Supplementary Data for a detailed analysis of the studies). Analysis that included measures such as the ICC demonstrates the reliability of a single ejaculate for referral to infertility investigation and treatment leading to the conclusion that examination of a single ejaculate is sufficient. This conclusion is consistent with ASRM and EAU recommendations. For example, the ASRM (2015a) state ‘that at a minimum, the initial screening…should include analysis of at least one semen sample’ and the EAU state that ‘if the results of semen analysis are normal according to WHO criteria, one test is sufficient’ (Jungwirth *et al.*, 2015). The question is whether there is a sub-group of men being investigated for infertility that require a repeat semen analysis. If so, which group would this be? It is most likely to be men with ejaculate results in between very good and very poor, i.e. those in the ‘intermediate’ range (Guzick *et al.*, 2001). Guzick *et al.* (2001) presented a model where patients were divided into three categories: poor, intermediate and good ejaculate results. Creating three groups for men undergoing infertility investigation may seem somewhat hypothetical. However, there is a considerable overlap in ejaculate analysis results from fertile and infertile men and the intermediate range corresponds largely to this mixed group. For men with results in the ‘intermediate’ group a repeat analysis could provide further information—confirming earlier results or pointing to a less or more severe problem. It is, therefore, logical to use a ‘borderline zone’ between good and very poor results (Guzick *et al.* 2001; Björndahl, 2011), and perhaps restrict repeat analysis to this mixed group.

However, a recommendation that analysis of a single ejaculate is sufficient to determine the most appropriate investigation and treatment pathway is a controversial strategy. In some cases, this appears contrary to conventional clinical practice where a plethora of well-documented variables are known to affect semen analysis thus potentially reducing the clinical value of a single ejaculate. Additionally, analyses of only one ejaculate is contrary to previous standard WHO recommendations. However, a primary reason for the variability in semen parameters is the failure of some laboratories to adhere to standard WHO recommendations (Mallidis *et al.*, 2012) and failure to control for key parameters e.g. abstinence, that increases the variability of the test. Importantly, the majority of these can be mitigated by adopting and adhering to appropriate practices and WHO procedures (WHO, 2010). Notwithstanding this, adoption of a strategy of analysis of a single ejaculate should be accompanied by a detailed cost-benefit analysis to examine if and at what stage additional semen assessment, particularly in the borderline zone, is appropriate. It is also important to emphasize that this recommendation applies only to referral for infertility investigation and treatment, and is not relevant, for example, if the aim of a study it to establish a ‘true’ value of sperm production or sperm output rate, for example, where a single ejaculate is not sufficient (Amann and Chapman, 2009).

In summary, we strongly recommend that based on a high quality of evidence that analysis of a single ejaculate is sufficient to determine the most appropriate investigation and treatment pathway although semen analysis could be repeated if one or more abnormalities are found (Table I).

There are a number of significant areas for future. First, for example, there is a need for large multi-centre studies to examine the predictive values in semen analysis to identify men likely to contribute to spontaneous pregnancy, ART pregnancy, fertilization failure, pregnancy loss/miscarriage, time to pregnancy (TTP) and live birth. Second, a fundamental problem with developing new therapies or diagnostic tests for male infertility is the limited understanding of the formation, maturation and physiological workings of the normal and dysfunctional spermatozoon. There is an urgent requirement to understand these cellular, molecular biochemical and genetic mechanism(s) in order to formulate appropriate diagnostic assays and rational therapy for the male.

### What key male lifestyle factors impact on fertility?

This is, and is likely to remain, a topical issue. The focus of the analysis was on obesity, smoking and heat exposure.

#### Does obesity influence semen parameters?

Obesity is a global health problem. It impacts not only cardiovascular diseases but also on many other related health disorders. Obesity may adversely affect male reproduction by endocrinologic, thermal, genetic and sexual mechanisms (Reis & Dias, 2012). As such, obesity must be considered as a potential causal factor in male infertility. However, two key meta-analyses published in this area show conflicting data (MacDonald *et al.*, 2010; Sermondade *et al.*, 2013) with the latter concluding: ‘overweight and obesity were associated with an increased prevalence of azoospermia and oligozoospermia’. Additionally, there are a number of cross-sectional and longitudinal studies (MacDonald *et al.*, 2013; Eisenberg *et al.*, 2014 and Andersen *et al.*, 2015) that reported some negative associations between semen parameters and obesity. A cross-sectional study of New Zealand males (2013) showed that morphology was the only parameter that correlated with BMI (MacDonald *et al.*, 2013). A longitudinal study of American males reported an increased OR for decreased ejaculate volume and total sperm count associated with obesity (Eisenberg *et al.*, 2014). Furthermore, the OR for lower sperm concentration and total sperm count increased with waist circumference. A cross-sectional study of Norwegian men found a significant decline in all standard semen quality markers with increasing BMI. BMI was also negatively associated with hormones of reproduction (Andersen *et al.*, 2015). A cross-sectional study of 4400 men attending infertility clinics in the USA reported a significant negative relationship between obesity and semen parameters (Bieniek *et al.*, 2016). Moreover, the incidence of azoospermia and oligozoospermia was more prevalent in obese men. Data from the CHAPS-UK study found no evidence for an effect of BMI on either motile concentration (Povey *et al.*, 2012) or sperm morphology (Pacey *et al*., 2014). The ASRM concluded in their ‘Obesity and reproduction: a committee opinion paper committee opinion’ paper in 2015 that ‘obesity in men may be associated with impaired reproductive function’ (ASRM, 2015b).

Based on the number of papers able to be included in each meta-analysis paper (*n* = 31 and 25 for MacDonald *et al.*, 2010 and Sermondade *et al.*, 2013, respectively) and the contradictory results, along with cross-sectional and longitudinal study outcomes, a reasonable conclusion is that additional well-controlled, population-based trials are necessary before stronger conclusions regarding the potential impact of obesity on semen parameters can be made. The methods used to assess obesity should also be standardized. Obesity studies should include measurement of reproductive hormones, as the only meta-analysis paper (MacDonald *et al.*, 2010) that included hormonal parameters concluded ‘There was strong negative relationship for testosterone, SHBG and free testosterone with increased BMI.’ In a recent cross-sectional study, (Andersen *et al.*, 2015) the correlation between the three hormones and obesity was affirmed. Studies on the impact of weight-loss nutritional interventions on reproductive health are missing. Bariatric surgical intervention reports exist, however the outcomes are mixed and access is often restricted based on socio-economic status.

In summary, the evidence supports a detrimental effect of obesity on many aspects of health, and evidence is conflicting about a potential effect on reproductive function. Therefore, we strongly recommend, based on a moderate quality of evidence, that males presenting for fertility evaluation should be counseled about weight-loss strategies when the BMI and waist circumference data demonstrate obesity and especially morbid obesity (Table I).

#### Does exposure to heat adversely affect semen parameters and/or male fertility?

Perhaps surprisingly, the quality of the evidence linking heat to human male infertility is relatively poor. Data from animals, primarily by experimental manipulation of the testis, strongly suggest an adverse effect of heat on spermatogenesis and subsequent fertility (Durairajanayagam *et al.*, 2015). Additionally, it is well established that cryptorchidism is associated with abnormal spermatogenesis attributable, at least in part, to heat exposure of the testes to core body temperature (Hutson *et al.*, 2013).

A key question is: When the testes are in the scrotum, does heat exposure adversely affect semen parameters and male fertility? Many studies link certain activities to increases in scrotal temperature (for example use of saunas, hot baths), but do not follow this up with information about the effect on semen parameters (Durairajanayagam *et al.*, 2015). Of the studies that do investigate the effect of temperature on semen parameters, fewer still provide indicators of fertility outcomes such as live birth rate or TTP. Only Thonneau *et al.* (1997) assessed TTP, and this study alone, with a small sample size, was insufficient to draw robust conclusions about the effect of heat exposure on fertility. The reported reduction in semen parameters caused by heat is often small and it is unclear what effect, if any, this would have on biological fertility. For example, Hjollund *et al.* (2000) reported a lower average sperm concentration in men with higher scrotal temperatures, but this was still in the normal range (above 15 × 10^6^/ml, WHO, 2010). Additionally, some studies did not measure scrotal temperature, attributing a difference in semen parameters to an activity without providing evidence that there was any rise in testicular temperature (e.g. a study of taxi drivers Figà-Talamanca *et al.*, 1996).

No RCTs were found and almost all of the data were collected retrospectively. Previous studies often suffer from small sample size (e.g. Garolla *et al.*, 2013) and confounding factors such as lifestyle factors (e.g. Figà-Talamanca *et al.*, 1996). Very few of the studies used control groups which further complicates interpretation. No systematic reviews or meta-analyses have been performed, which is probably because of the significant variation in study design. The available studies use different ages of participants, different outcomes, subjective definitions of heat exposure, and even different criteria for semen analysis (Zorgniotti & Seaflon, 1988 and Cherry *et al.*, 2014).

Some studies investigated the effect of occupational heat exposure on male fertility, for example welders. The evidence that this activity affects male fertility is low, and extreme heat exposures such as this do not represent the normal heat exposures of the general population (caused by wearing different types of underwear, sedentary position etc.). Povey *et al.* (2012) and Pacey *et al.* (2014) concluded that there was no significant effect of lifestyle factors, including heat exposure, on semen parameters, and others also failed to find a significant effect, for example, Støy *et al.* (2004) and Eisenberg *et al.* (2015), although Priskorn *et al.*, (2016) did report a negative association between watching television for 5 h/day and sperm concentration but there was no measurement of heat exposure.

Suffice it to say that further work is required to elucidate the effects of heat exposure on male fertility. Ideally, scrotal temperature, semen parameters and a measure of fertility outcomes, such as live births, would have to be measured in prospective cohort studies. Studies must use comparable measures of semen analysis, methods for measuring scrotal temperature and definitions of infertility in order for comparisons to be made. This would allow a systematic review or meta-analysis of the evidence to be carried out.

In summary, we strongly recommend, based on a very low quality of evidence, that there is insufficient evidence to conclude that exposure to heat, be it occupational or as a result of clothing or body position, affect semen quality and/or male fertility (Table I).

#### Does cigarette (tobacco) smoking adversely affect semen parameters and/or male fertility?

Most of the published literature on cigarette (tobacco) smoking and male fertility only looks at the effect of smoking on semen parameters. The quality of the recent evidence linking cigarette smoking to decreased semen quality is moderate, as a systematic review and meta-analysis of 46 cross-sectional studies (Li *et al.*, 2011) found that smoking was associated with reductions in all of the semen parameters. A further meta-analysis of the literature since 2010 analysing 20 studies with 5865 participants (Sharma *et al.*, 2016) also concluded a significant negative effect of cigarette smoking on all semen parameters. One prospective cohort study was identified which examined the effect of smoking status on semen quality (Yang *et al.*, 2015). This study reported a significant reduction in total sperm count (*P* = 0.012) and concentration (*P* = 0.023) after multivariate analysis. Several cross-sectional and case-control studies found differing effects of smoking on semen quality. For example, Jeng *et al.* (2014) reported a decreased proportion of sperm with normal morphology in those who smoked >10 cigarettes/day (*P* = 0.04), whilst Povey *et al.* (2012) found no significant effect of smoking on motile sperm concentration and Pacey *et al.* (2014) found no significant on sperm morphology. Several studies reported an association between smoking and changes in blood hormone levels. For example, Jeng *et al.* (2014), Al-Matubsi *et al.* (2011) and Lotti *et al.* (2015) all reported increased serum testosterone in smokers compared to non-smokers.

The definitions of smoking status vary between studies, with some studies failing to define which participants they classed as ‘smokers’ (Caserta *et al.*, 2013) and some studies using cotinine levels to evaluate smoking status rather than pack-years or number of cigarettes smoked per day. Lotti *et al.* (2015) considered those that had smoked for <1 year to be ‘never smokers’. The studies also varied on how they dealt with ex-smokers—in some studies only those who currently smoked were ‘smokers’ and in others, anyone who had exceeded 1-pack-year at any time in their life were ‘moderate smokers’ (Anifandis *et al.*, 2014). In some cases, smoking status was divided into light, moderate and heavy, and again, these definitions were inconsistent. These inconsistencies made it challenging for Li *et al.* (2011) to establish a dose-dependent trend of smoking and reduced semen quality. Much of the current evidence comes from men presenting to infertility clinics, and may not represent the effect of smoking on semen quality and/or fertility in the general population. Also, smoking status was generally self-reported. This could introduce bias to the evidence based.

There are few studies that examine the effect of smoking on indicators of fecundity, such as TTP. One retrospective cohort study, Mutsaerts *et al.*, (2012) found that paternal smoking had no effect on TTP. ASRM (2012a) concluded that there was insufficient evidence of the association between smoking and male infertility, despite the fact that smoking has been shown in many studies to affect semen quality, however, a review of this recommendation is due to be published in the near future.

Systematic review and meta-analysis are required to investigate the effect of smoking on blood hormone levels, and of measures of fecundity, such as TTP. However, this evaluation would require a greater number of well-designed, prospective cohort studies with consistent definitions of smoking status. The participants could be stratified into levels of smoking, which could be assessed by biochemical methods to reduce self-reporting bias. The outcomes should include measurement of semen parameters as well as measures of fecundity and fertility, such as TTP and live birth rate.

In summary, we strongly recommend based on a moderate quality of evidence that there is some evidence to suggest a negative effect of cigarette (tobacco) smoking on semen quality/male fertility but not all studies report this. However, as smoking has an adverse effect on general health and wellbeing it is recommended that men trying for a pregnancy should abstain from smoking (Table I).

As stated previously the effect of lifestyle (and environmental factors) affecting male infertility is likely to remain a rapidly developing and topical issue. There are a large number of significant areas for future research. Primarily large-scale prospective multi-centre trials encompassing a variety of geographical locations are required to examine the effects of lifestyle (e.g. obesity, heat exposure, smoking, recreational and medical drug use, high-intensity sports) and environmental factors (e.g. occupational exposures, endocrine disrupting chemicals (EDCs)/toxins) on spermatogenesis, semen analysis, male fertility and fertility outcomes, including the health of subsequent offspring.

### Do supplementary oral antioxidants and herbal therapies significantly influence fertility outcomes for infertile men?

There is a significant body of data to support the concept that oxidative stress plays a key role in sperm dysfunction and male infertility (Aitken *et al.*, 2014). Consequently, antioxidant treatment of the infertile man may improve semen quality and/or fertility. However, the key question is: Are there data to support this? To address this question two approaches were used: use of a recent Cochrane review (updated; Showell *et al.*, 2014); and primary analysis including analysis of the literature on herbal therapies.

The Cochrane review (Showell *et al.*, 2014) examined the use of antioxidants. Forty-eight published studies were included in the systematic review with 37 studies included in the meta-analysis. In summary, only 7/48 trials reported on clinical pregnancy rate, only four of which went on to report live births. The authors recommended that until live birth and clinical pregnancy rate are robustly reported by all infertility trials, it is not possible to draw clear conclusions on the use of antioxidants for infertile men. Additionally, they concluded that the low-quality evidence from only four small RCTs suggested that antioxidant supplementation in infertile males may improve live birth rates. Data were lacking on other adverse effects. Importantly they suggested that ‘further large well-designed randomized placebo-controlled trials are needed to clarify these results’. Our primary analysis of the literature came to a similar conclusion as the Cochrane review (Showell *et al.*, 2014) as there were very few additional studies examined (e.g. Raigani *et al.*, 2014).

Far fewer studies examined the use of herbal therapies (Y virilin, Saffron, Addyzoa). Two-thirds of the studies showed some improvement in semen parameters and one study reported a positive effect on sperm membrane integrity (Omu *et al.* 1998). Two studies showed improvements in sperm DNA integrity after herbal therapy (Omu *et al.* 2008; Raigani *et al.* 2014). After the use of herbal therapies only one of three (33.3%) studies reported a positive influence on pregnancy rates. Adverse effects were reported in 16.6% of the studies and most of them were mild to moderate. However, it is worth noting the study of Safarinejad *et al.* (2011) within which a large number of patients had adverse haematological reactions during treatment with the compound Linn Crocus sativus (Saffron).

An inevitable conclusion was that the methodological quality of most studies in the literature on antioxidants and herbal therapies to treat male infertility is poor. Additionally, the heterogeneity of the selected studies makes meta-analysis challenging. A further complication is that techniques to measure oxidative stress, antioxidant capacity and/or DNA damage are not standardized between all the trials. Moreover, there is often a lack of clear pre-selection of a sub-group for testing e.g. confirmed high-reactive oxygen species/DNA damage, reduced antioxidant capacity. Taking these factors into account, oral antioxidant therapy may improve seminal oxidative status in infertile men either by decreasing oxidative stress or by increasing the total antioxidant capacity but the evidence is of poor quality. In some cases, positive relationships are manifested in improvements in semen parameters, most often sperm motility. This may explain the higher pregnancy rates after antioxidant therapy compared to placebo but further detailed studies are required. Studies evaluating the supplementation with herbs constitute only a small part of the available literature. For these studies, the heterogeneity of the trials do not allow a robust conclusion to be drawn. Suffice it to say, there are no high-quality data to support the use of a single antioxidant or a specific combination of antioxidants. Further, it is not possible to recommend an effective treatment regimen.

In summary, we strongly recommend based on low-quality evidence that there are insufficient data to recommend the use of supplemental antioxidant therapies for the treatment of men with abnormal semen parameters and/or male infertility. Additionally, we strongly recommend based on very low quality of evidence that there are insufficient data to recommend the use of herbal therapies for the treatment of men with abnormal semen parameters and/or male infertility (Table I).

There are significant areas for future research; there is an absolute and urgent requirement for large, well-designed placebo-controlled randomized trials with primary outcomes of TTP and live births (including health of these births) reported in well-characterized groups to examine, for example, the effects of dietary supplementation, vitamins and herbal remedies.

### What are the evidence-based criteria for genetic screening of infertile men?

The determination of whether an infertile male benefits from having a genetic evaluation depends on the aetiology of the reproductive compromise and the severity. A detailed history and comprehensive PE, coupled with adjunctive tests, such as a semen analysis, hormone assays and on occasion testis biopsy, help clarify to which diagnostic category the patient belongs and, as a helpful consequence, assists in determining which genetic studies may be fruitful. For example, if a male has a reproductive history consistent with a known cause of resultant spermatogenic failure, such as chemotherapy, bilateral mumps, orchitis with resultant atrophy or current use of anabolic steroids, and is presently severely oligozoospermic or azoospermic, it can be assumed that these are the proximate reasons for the reduced/absent spermatogenesis and no genetic evaluation needs to be undertaken. The focus of the recommendations was on Karyotype, Y micro deletions and cystic fibrosis (CF) mutation analysis.

#### Y-chromosomal microdeletions and karyotype in men with spermatogenic dysfunction

No Cochrane reviews were identified. The primary evidence was from studies by Rozen *et al.* (2012), and Krausz *et al.* (2014) and practice statements from ASRM (2012b), AUA (2011) and EAU (Jungwirth *et al.*, 2015). Based upon the evidence, we recommended that in men who have a history, PE and hormonal assays consistent with severe oligozoospermia or non-obstructive azoospermia (NOA), both a karyotype and Y-chromosomal microdeletion assay should be offered.

*Karyotype*: Men with a sperm count <5 million/ml show a much higher rate of autosomal abnormalities than fertile populations (around 4%) while the highest frequency is found in NOA men (mostly Klinefelter syndrome). Klinefelter syndrome [47,XXY including variants (48,XXXY), and XX males (SRY+ and SRY−)] is the most common of the sex chromosomal aneuploidies. Translocations may be found in a relatively small percentage of men with severe oligozoospermia and azoospermia (Yatsenko *et al.*, 2010). The benefits of knowing if there is a chromosomal abnormality are in the planning for therapy and in the future follow up of the patient. As such, karyotype analysis should be performed prior to either use of ejaculated sperm in conjunction with ICSI, or prior to operative testis sperm extraction (TESE). The *a priori* knowledge of a chromosomal translocation, depending upon its exact nature, may significantly alter the thought process and therapeutic strategy of an upcoming ICSI cycle by employing PGS to allow the transfer of only balanced or normal embryos while discarding those that are chromosomally unbalanced (e.g. Dul *et al.*, 2012).

In summary, we strongly recommend, based on a high quality of evidence, that karyotype testing should be performed on all males with severe oligozoospermia (<5 × 10^6^/ml) or NOA prior to any therapeutic procedure (Table I).

*Y-chromosomal microdeletion assay*: The molecular geography of the Y chromosome is such that microdeletions (not recognizable by cytogenetic methods) may occur that partially or completely eliminate the azoospermia factor (*AZF)a* or the *AZFb/c* region from the genome, and, consequently, any important ‘spermatogenic necessary’ genes that reside in those intervals. Frequency data compiled by Rozen *et al.* (2012) show an overall incidence of Y microdeletions in the *AZFc* region in 1/27 men, which varied depending on the Y haplotype. The importance of Y microdeletion testing in the severely oligozoospermic or azoospermic male prior to any therapy (ICSI using ejaculated sperm or TESE) is for prognosis and consideration of PGD. For example, data show that when a complete *AZFa*, *AZFb* or *AZFb/c* microdeletion is present (~1–2% incidence of each in the NOA man) no spermatozoa will be found on TESE. When there is no possibility that sperm will be present, it is unhelpful and hurtful for the male to be operated upon and, in the latter circumstance, for the female partner to have an ovarian stimulation unnecessarily. Men with *AZFc* microdeletions can produce spermatozoa that are capable of fertilization, embryo development and term pregnancy (Oates *et al.*, 2002). An *AZFc* microdeletion results in a quantitative reduction in spermatogenesis with maintenance of spermatozoa quality and function. All males born will directly inherit the *AZFc* microdeletion.

In summary, we strongly recommend, based on a high quality of evidence, that Y chromosome microdeletion (YCMD) testing should be performed on all males with severe oligozoospermia (<5 × 10^6^/ml) or NOA prior to any therapeutic procedure (Table I).

#### CF-mutation analysis in men with Congenital Bilateral Absence of the Vas Deferens or clinical CF

No Cochrane reviews were identified. The primary evidence was from studies by Yu *et al.* (2012), Xu *et al.* (2014) and Lommatzsch and Aris (2009), and practice statements from ASRM (2012b), AUA (2011) and EAU (Jungwirth *et al.*, 2015).

Men with clinical CF (pulmonary and pancreatic dysfunction) will also have absence of the vasa and seminal vesicles bilaterally and will, consequently, have a low volume, low pH, and an azoospermic ejaculate. The incidence in males of northern European heritage is 1:2000. An equal frequency of men with low volume, acidic pH, azoospermia will have congenital bilateral absence of the vas deferens (CBAVD) with little respiratory or pancreatic disease, the vast majority of whom will possess mutations/pathogenic abnormalities in both maternal and paternal CF alleles. Whether one presents with respiratory tree (including sinuses) and/or pancreatic disease, simply absence of the vas deferens or somewhere clinically between these phenotypic extremes depends upon exactly which mutations/abnormalities in the alleles are inherited. There are >1985 recognized mutations in the CF transmembrane conductance regulator (*CFTR*) gene (Cystic Fibrosis Mutation Database: The Hospital for Sick Children, Genetics and Genomics Biology. Toronto: 1989 [Accessed: August 2014]. Available at: http://www.genet.sickkids.on.ca/cftr/app.) The *CFTR* gene has 27 exons spanning 250 kb of chromosome 7 (7q31) and encodes an mRNA of 6.5 kb and the final protein contains 1480 amino acids. Certain mutations, such as c.1521_1523delCTT (legacy name: F508del), severely impair either quantity or functional quality of the CFTR protein determined by that allele. Other abnormalities, such as the 5 T polymorphism in intron 8 (5 T), only mildly impair quantity or functional quality of the CFTR protein determined by that allele. It is the combination of the two that correlates to the severity of disease expression. If a person is homozygous for c.1521_1523delCTT, for example, s/he will have problematic respiratory and pancreatic disease manifested and diagnosed in childhood. However, if a male has inherited the ‘5 T’ allele and c.1521_1523delCTT on the opposite allele and is therefore a compound heterozygote, pulmonary and pancreatic function may be clinically normal and CBAVD is the only recognizable phenotypic consequence. Bilateral vasal absence, then, is the most sensitive indicator of a biallelic CF gene abnormality as there is differential expressivity and sensitivity to CFTR in different epithelial tissues. In addition, it has been postulated that severity of phenotype may be modified by polymorphisms in unrelated genes such as TGFB1 (transforming growth factor) and EDNRA (endothelin receptor type A) (Havasi *et al.*, 2010). In a recent meta-analysis by Yu *et al.* (2012) of CBAVD patients, 78% had at least one mutation identified, 46% had two mutations identified and 28% had only one mutation identified. The most common heterozygous mutation pairing was F508del/5T (17% of CBAVD cases) and F508del/R117H (c.350G > A; 4% of CBAVD cases). The poly-thymidine tract in intron 8 has three alleles consisting of 5, 7 and 9 thymidines that are found in 5, 85, and 10%, respectively, of the general population. In the presence of 5T, there is reduced splicing of Exon 9 and, as a consequence, reduced expression of full-length CFTR. Because 5T acts as a ‘mutation’ when trans (on the opposite allele) to a defined *CFTR* mutation, e.g. F508del, the poly-T tract in intron 8 must be defined in cases of CBAVD. Poly-T tract analysis is often only a reflex assay when R117H is detected (Chen and Prada, 2014). However, many of the studies in the meta-analysis of Yu *et al.* (2012) were conducted in the early years after discovery of the association of CBAVD and CF mutations, when only a small cohort of mutations was known and searched for (Anguiano *et al.*, 1992). The more comprehensive the assay, the more patients will have their second abnormality identified. Although a *CFTR* mutation genetic basis underlying most cases of CBAVD was a statistical certainty, this meta-analysis provides well-defined summary values which, in all likelihood, will be modified upwards in future years as even more complete *CFTR* assessment is accomplished for men with CBAVD.

The distribution of *CFTR* mutations and polymorphisms differs depending upon the ethnic/geographical origin of the patient/population being studied. As reviewed by Lommatzsch and Aris (2009), F508del is the most common mutation leading to CF worldwide but varies in its frequency based upon ethnicity/geographical location: 70–80% in CF patients from northern Europe, 50% in CF patients from southern Europe, 48% in African Americans, 46% in US Hispanics, 30% in Ashkenazi Jews, 18% of Tunisian CF patients and rarely in Native Americans. Furthermore, in the Ashkenazi Jewish population c.3846G > A (legacy name W1282X) is the most common mutation found (48% frequency). The meta-analysis by Xu *et al.* (2014) which specifically looked at F508del, 5T, and M470V, supports the above findings, concluding that there are significant associations between F508del and CBAVD (*P* < 0.001, OR = 22.20, 95% CI = 7.49–65.79), 5T and CBAVD (*P* < 0.001, OR = 8.35, 95% CI = 6.68–10.43).

In situations of CF or CBAVD, it is always necessary to screen the female partner for CF gene abnormalities so as to have a proper assessment of the risks of any offspring inheriting one of the two paternal mutations and the maternal mutation and presenting with clinical CF or, at the least, CBAVD if male. In addition, the benefit of testing the male with CBAVD helps provide information for his siblings who have a 75% chance of harbouring at least one (or possibly both) of the mutations inherited by the patient from his parents. Finally, patients may have mild CF symptoms such as ‘sinusitis’ or ‘bronchitis’, not previously recognized to be CF-mutation related and which, with a full understanding of their genetic basis, may be therapeutically managed in a different fashion. Not all CBAVD appear to be caused by/associated with *CFTR* mutations and abnormalities, and these cases may be secondary to a distinct genetic aetiology that affects mesonephric duct development. The phenotypic end-product may be CBAVD and unilateral renal agenesis, as described by McCallum *et al.* (2001). Therefore, in cases of CBAVD where no *CFTR* mutations are identified, renal ultrasonography is indicated.

In summary, we strongly recommend based on the high quality of evidence that appropriate *CFTR* mutation analysis should be offered to all males with CBAVD or CF (Table I).

There are significant areas for future research in the genetic screening of the infertile male. For example, what are the long-term health outcomes of children born from infertile men, can cost-effective tools for genetic screening in men (karyotype, Y micro deletions and CF-mutation analysis) in low-income settings be developed, and what is the genetic basis of unilateral absence of the vas associated with unilateral renal agenesis?

### How does a history of neoplasia and related treatments in the male impact (his and his partners) reproductive health and fertility options?

In a number of aspects this was a very challenging question to address. Although there were several reviews in the area, for example Tournaye *et al.* (2014) and Samplaski and Nangia (2015), and key recommendations from national societies, for example American Society of Clinical Oncology (ASCO) (Loren *et al.*, 2013), the therapeutic agents and treatment regimens are continually evolving. Additionally, there were limited data on key aspects of the question, such as advice on the contraception window post-treatment and health of the offspring of both juvenile and adults cancer survivors.

However, on a general level, there is consistency in the recommendations of the major medical societies in Europe and USA: the European Society for Medical Onclology (Peccatori *et al.*, 2013) and ASCO (Loren *et al.*, 2013). For example, storage of semen samples is the primary option to potentially preserve fertility of men (and boys producing sperm in the ejaculate) who are undergoing chemo/radiotherapy regimes (Loren *et al.*, 2013; Peccatori *et al.*, 2013). As such, the overwhelming evidence suggests that all patients should be provided with information about the impact of their cancer treatment on spermatogenesis and the option of sperm banking. Whether regimens carry a high or lower likelihood of long-term fertilty impairment, given the variability in individual response to treatment and the potential for relapse, the evidence would recommend that sperm cryopreservation should always be considered and services be available and affordable.

Counselling should also include the fact that there is little chance of recovery from azoospermia after 10 years following radiotherapy (Sandeman, 1966), total body irradiation (Rovó *et al.*, 2006) or chemotherapy (Meistrich *et al.*, 1992, Heikens *et al.*, 1996), however, contraception should continue to be considered if paternity is not desired. Actively attempting pregnancy during cancer treatment must be avoided, however, if an accidental pregnancy would occur during cancer treatment this should not automatically be considered an indication for elective termination. The pregnant couple should be offered counselling and appropriate foetal diagnostic interventions, for example, minimally an evaluation of the foetus through ultrasound (De Santis *et al.*, 2008).

Although the fertility of male cancer survivors is reduced, registry data have identified subgroups dependent on cancer type, age at onset, treatment modality and dose, whose fertility is not different from the general population (Tournaye *et al.*, 2014). Pregnancy outcomes, such as pre-term delivery, low-birth rate and miscarriages, seem to be comparable to the general population but there are conflicting data regarding the risk of malformations (Ståhl *et al.*, 2011). Generally, a co-ordinated approach between the healthcare professionals involved in cancer treatment and the reproductive medicine specialists is highly recommended although regrettably not always achieved.

In summary, we strongly recommend that based on moderate quality of evidence that every male cancer patient should be provided with information about the impact of his cancer treatment on spermatogenesis and the option of sperm banking. Additionally, we strongly recommend based on low quality of evidence that: patients should be advised to use contraception if they do not wish to procreate even after prolonged periods of azoospermia following radiotherapy, as recovery is possible; male cancer patients should be informed that pregnancy outcomes in partners are good but a slightly higher risk of congenital anomalies in their offspring cannot be excluded (Table I).

There are significant areas for future research. For example, there are insufficient data to advise men regarding the contraception window post-treatment and a lack of systematic data on the risk of birth defects following accidental conception during treatment. It also has to be determined if there are any effects in second generations of cancer survivors following cancer treatment; there are no data regarding the risk to partners or offspring from chemotherapeutic agents in semen.

### What is the impact of varicocele on male fertility and does correction of varicocele improve semen parameters and/or fertility?

For this question, and after discussion with this working group of experts and with the leads of the WHO GDG, a recommendation was made to use the Practice Committee of the ASRM; Society for Male Reproduction and Urology (2014). Report on varicocele and infertility: a committee opinion (evidence level IV). As the methodology used to construct this report was not equivalent to the WHO GRADE assessment, the conclusions cannot be suggested as a recommendation but as an opinion based on a review of the literature. As such these are suggested as Good Practice Points, namely:

- Treatment of a clinically palpable varicocele may be offered to the male partner of an infertile couple when there is evidence of abnormal semen parameters and minimal/no identified female factor, including consideration of age and ovarian reserve.
- IVF with or without ICSI may be considered the primary treatment option when such treatment is required to treat a female factor, regardless of the presence of varicocele and abnormal semen parameters.
- The treating physician's experience and expertise, including evaluation of both partners, together with the options available, should determine the approach to varicocele treatment.

An analysis by Shridharani *et al.*, (2016) presents a detailed assessment of the EAU, ASRM and AUA recommendations on varicocele. The differences in their recommendations and the complexities of conducting a long-term prospective trial that would definitively answer this question clearly indicate that significant further research is necessary to guide clinical management.

## Challenges and future research opportunities

It was perhaps an inevitable conclusion that, in conducting these analyses, gaps in the literature would be identified. It would be surprising if this was not the case. However, what was surprising was the substantial nature of the gaps where effectively little or sometimes no research had been performed. Of additional note was the sometimes low quality of the available evidence. Such a combination makes formulating informed evidence-based decisions for the diagnosis of the male difficult (see Table I for summary). However, conversely, a number of issues were identified with clear and significant opportunities for the way forward.

### Key themes in developing these recommendations

#### Areas for research focus

We present key areas for research focus that demand investigation. Overall one high priority area for research was to gain a better understanding of the production, formation and workings of a human spermatozoon. There is an urgent requirement to understand these cellular, molecular biochemical and genetic mechanism(s) in order to formulate appropriate diagnostic assays, develop rational therapy for the male, and understand how external factors, such as the environment, negatively or positively influence these processes. Not surprisingly, this is not unique to our discipline. For example, a new strategy to understanding neurodegeneration with a primary focus on the formation and function of the cell is now strongly advocated as absolutely essential to accelerate progress for understanding neurodegenerative disorders (Kosik *et al.*, 2016). Although research in understanding the workings of the human spermatozoon has progressed significantly over the last 10 years, there is still the need to catch up and then keep pace with the knowledge base in other cellular systems. It is unlikely that a series of robust diagnostic tools for sperm function can be developed without further detailed understanding of the working of the normal and dysfunctional cell. Additionally, without this knowledge, the complementary development of a drug(s) that a man can take or have added to his spermatozoa *in vitro* to improve sperm function will continue to remain elusive. Premature introduction of putative but unproven diagnostic and/or therapeutic tools into clinical practice in ART can hinder rather than advance progress in the field for the long-term (Harper *et al.*, 2012, 2017; Spencer *et al.*, 2016).

An additional high priority theme area for research was to examine the long-term health outcomes of the children born from men with compromised fertility (including those who may also have been treated with ART) whatever the nature of the compromising event(s) (e.g. genetics, environmental, iatrogenic and/or occupational). Moreover, this analysis needs to assess the effects in various geographical locations. In addition to the research themes which accompany each question, we identified other areas that require addressing. For example, what is the impact of age on male fertility? What are the underlying causes related to the male for IVF–ICSI treatment failure? Which gene (or epigenetic) defects in men can predict ART outcomes? What are the most effective educational initiatives towards improving understanding of male infertility? What are the attitudes of men and women (in various geographical locations) towards the investigation of male fertility? Certainly, in presenting this analysis, a plethora of research questions has been generated.

#### Requirement to obtain robust data

A consistent theme was the focus on the quality of evidence available to support any recommendations. The criteria used can be presented in two, not mutually exclusive, formats: Traditionally, the quality of evidence is represented by using a range of different levels. However, it is clear that a considerable degree of the research related to the diagnosis of male infertility does not easily fit into these categories. The evidence is sometimes observational and not easily amenable to meta-analysis. With respect to the scientific and clinical evaluation of diagnostic methods i.e. methods that measure characteristics of the individuals, RCT are not usually appropriate. Primarily there is no inclusion of a separate group to be used as a control i.e. a group not obtaining the investigated treatment. In the evaluation of a diagnostic method, the control of the diagnostic method can be obtained with another independent method, or from the final clinical outcome. From this point of view, the highest level of evidence cannot easily be obtained through a RCT.

Where data/evidence were available the quality of the evidence was often judged to be sub-optimal. One issue was the robustness of studies and thus general applicability. The robustness of the evidence base in reproductive medicine has been discussed (Evers, 2013, Barratt, 2016a, 2016b; Glujovsky *et al.*, 2016) and is of course not just a theme in reproductive medicine (Baker, 2016). Robust methods must be developed and subsequently utilized. One example where we have perhaps made relatively little progress is the technical challenges of semen analysis. However, with a greater appreciation of the difficulties in this arena (Carrell and De Jonge, 2016) now is perhaps the time for the WHO to produce a 6th version of the semen assessment manual. The evidence based for the current (5th version) manual is at least 10 years out of date and a lot has changed. Nevertheless, there are areas of good practice: European Academy of Andrology guidelines and recommendations for quality control of the Y-chromosomal microdeletion assay (Krausz *et al.*, 2014) are commonly used. Robustness also applies to the verification of key recommendations from, for example, professional societies (see Ventimiglia *et al.*, 2016). In the future we need to heed the lessons of the past and make sure the evidence base, including the use of robust tools, is significantly improved.

#### The theme of international studies

Male infertility is a global and significant health problem. A consistent theme is the requirement for national and international efforts with large-scale, multi-centre studies encompassing different geographical locations. Considerable regional variations in key indices of male reproductive health have been reported (Skakkebaek *et al.*, 2016) but these are often on a relatively local scale. It is critical to understand potential variations in sentinel markers of male reproductive health, in other countries/regions as well as in low and middle resource settings globally, in order to inform on further policy and practice.

#### Context of resource setting

A key theme is the question of global implementation of diagnostic tools and therapeutic procedures, and especially in low-resource settings. Basic procedures do require resources and some recommendations although very simple procedures (e.g. sperm banking prior to potentially sterilizing cancer therapy) may be feasible in most settings but unfortunately they may not be in some areas without specialized storage facilities.

## Overall summary: the road to a healthy future for male infertility

There are significant advances in male reproductive health from developments on *in vitro* spermatogenesis to dissecting the workings of the mature spermatozoon. However, progress in this arena is comparatively slow. The robustness of the data is sometimes wanting, and thus the ability to provide the strongest evidence-based guidelines has proven to be a challenge. We provided recommendations based upon the evidence available. However, when looking to the future there is a need to understand why it has happened that the data are limited and not forthcoming, in order to inform the direction of future initiatives and endeavours that could result in more studies that yield robust evidence from men residing in high-, middle- and low-income country settings.

### What is the scale of the problem of male infertility and its consequences?

It is fundamental to know the prevalence of a disease in order to provide resources, estimate impact, make effective health economic arguments, present rational research questions and manage patients. However, there is a paucity of clinical data on the scale of the reproductive health problems and infertility in men on a global level, across all health economies. The best estimates on infertility from demographic health studies use the heterosexual woman, as she self-reports a problem with becoming pregnant, which further assumes a diagnosis of female infertility (due to minimal if any reporting of clinical diagnosis of the female), or possibly, an assumption of an infertile relationship; and, coupled with an even larger paucity of male diagnosis and management of infertility in low- and middle-income countries, any male infertility prevalence values based upon demographic health reports or from small private clinics are, at best, greatly extrapolated and highly inaccurate (Mascarenhas *et al.*, 2012). As a consequence, there is limited information on fundamental key markers such as the socio-economic impact that infertility and other diseases or disorders may have on the individual and society as a whole. Emphasizing the importance of the engagement of the male partner in the assessment of the infertile couple and the education of the public about male infertility should improve the care of the couple and expand our knowledge of the scale of the problem.

Moreover, there is an urgent need to determine the potential consequences of male infertility. This is a wide area of investigation which should extend beyond the confines of the couple and their immediate relationships to investigate, for example, the relationship of infertility with other diseases and what impact this has on the disease. There is increasing evidence (albeit currently primarily in animals) of the potential transmission to the next generation of damaging insults to the male germ line by epigenetic mechanisms (Siklenka *et al.*, 2015). This may become especially relevant with the effect of significant potential changes in the external environments on the fidelity of the germ line, and also the likely future potential use of more immature (Tanaka *et al.*, 2015), and even *in vitro* generated, gametes.

### Funding the science to match the scale of the problem

The lack of data in key areas reflects the overall paucity of high quality long-term national and international funding streams to support reproductive medicine, and male infertility in particular. Whilst this has been discussed previously it remains a fundamental block to further progress and a critical issue (Evers, 2013; Barratt, 2016a, 2016b). The WHO has not published evidence-based guidelines in this area, and the previous infertility/fertility manual for management of the infertile male based upon then current practice, was published in 1992. This effectively means ~25 years without an update on best practice and never any official guidelines for adoption and adaptation at country level. This is an unacceptable situation as the lack of universal international guidelines does not help advance the field of male infertility or male reproductive health in general. However, it is anticipated that these new evidence-based guidelines for the male will act as a platform for studies helping to raise the profile of male reproductive health.

Fundamentally, it is necessary to make more robust arguments to national and international agencies to help drive the research agenda and subsequently place reproductive medicine, with demonstrated impact on couples and individuals, at the vanguard of the funding landscape. The coupling of EDCs and male reproductive health may provide an example. In this field strong scientific, socio-economic healthcare and political arguments have consistently been marshalled to support research into what has been termed an ‘epidemic’ of male reproductive health problems (Hauser *et al.*, 2015; Skakkebaek *et al.*, 2016; Trasande *et al.*, 2016). The burden of the disease (via EDC) has been estimated and an impact assessment made (e.g. Olsson, 2014; Hauser *et al.*, 2015). For example, it is estimated that the EDC may contribute substantially to male reproductive disorders and disease equivalent to a staggering €15 billion cost in the European Union (Hauser *et al.*, 2015) although these figures are subject to considerable debate (e.g. Olsson, 2014; Woodruff, 2015). This type of analysis needs to be developed and continually fine-tuned for male reproductive health as a whole if additional support for the basic science and clinical base of male infertility are to be realized. We also need to realize that this support needs to encompass educating future students (some of whom will be consumers and others leaders in the field) by stressing the importance of studying reproductive health in the school/further education system (Finney and Brannigan, 2017). Developing and marshalling arguments to decision makers are a complex and continuous challenge. Male reproductive health needs firstly a series of hard-hitting documents providing an evidence based for the importance and challenges in our discipline. However, this is only the start of the process as delivery on these recommendations requires a myriad of discussions with international agencies, politicians and key decision makers. Evidence alone does not determine action—as exampled by the challenges in implementing regional, let alone global, policy to deal with the increasing CO_2_ in the atmosphere (Malakoff, 2017). We need to marshal a whole series of skills to effect policy, and scientific evidence on its own is insufficient for major policy changes (see Cairney, 2012, 2017). Stating the obvious—evidence-based guidelines require high-quality evidence, which will only be achieved with appropriate funding that will result in obtaining that data. Without robust data from all segments of society, the status of male reproductive health will remain invisible and the reproductive health needs of men will remain neglected into the foreseeable future.

## Supplementary Material

Supplementary Data
