# Supplementary material for: The diagnosis of male infertility: an analysis of the evidence to support the development of global WHO guidance—challenges and future research opportunities
Source: Hum Reprod Update. 2017 Jul 19;23(6):660–80. doi: 10.1093/humupd/dmx021 (PMC5850791; doi:10.1093/humupd/dmx021)
Supplement: Supplementary Data [file dmx021supplementarydatafinal.docx]

**Supplementary Data**

**Is single ejaculate assessment sufficient to categorize patients for further investigation and treatment? Primary data summary.**

*Background.*

There are many publications showing a high degree of variability between different ejaculates from the same individual; from such data stems the recommendation for repeated semen analysis in order to obtain a “true” result for the individual patient. Furthermore, it is essential to bear in mind that poor precision in ejaculate analysis can, if techniques are not well controlled, contribute significantly to variation. In contrast to the studies on variability, the aim of this analysis was to evaluate the possible correlation between ejaculates from the same individual. The objective was to determine if a second ejaculate analysis is likely to contribute significant additional information that cannot be obtained from the first ejaculate analysis. The comparison is between two ejaculates from the same individual. For the usefulness (generalization) of the results it is of interest that data are obtained from healthy individuals as well as from different patient groups. The preferred outcome would be that the results of the repeat analysis correlate well with the first analysis –there is a high reliability in the results and at least for very good and very bad results, respectively, a repeat analysis will not contribute significant new information.

*Search Strategy*

Literature searches were performed (Controlled February 1, 2017) at PubMed MEDLINE (www.pubmed.org). Searches were limited to studies published after January 1^st^ 2000 (see main text) until December 31, 2016, primarily due to the presumed advances in quality in laboratory andrology.

The systematic literature search was performed using a combination of the following keywords: Variability Semen Analysis; *Species:* Human; *Period:* 2000-2016 and resulted in a total of 107 articles. Selection of posts based on titles and abstracts reduced the number of included articles to six, of which three remained after reading the entire publications (elimination was because the articles only reported variability of single ejaculate analysis).

A second search was performed, based on articles similar to previously known publications e.g. by Stokes-Riner and co-workers (Stokes-Riner *et al.* 2007) which gave a further 64 posts that unfortunately were not useful as all studies fulfilling the search criteria had already been identified in the earlier search. Therefore a third search strategy was employed, using references in the previously accepted articles, resulting in two additional articles.

Studies were eligible if they presented primary data concerning the usefulness of repeat analyses – e.g. intraclass correlation coefficient (ICC), where an ICC close to 1.00 indicates high reliability between a pair of assessments.

*Results*

In total five articles gave information that can be used to support or reject a recommendation to base a decision of further infertility investigations of a man on a single semen analysis.

| **Reference** | **N** | **Conclusion** |
| --- | --- | --- |
| Francavilla *et al*. 2007.  Retrospective, IUI | 436 | Conc. ICC 0.92, 95% CI: 0.91–0.93; motile sperm conc. ICC 0.91, 95% CI: 0.89–0.92; rapid forward motility ICC 0.78, 95% CI: 0.75–0.81). “within-subject fluctuations are small relative to the between-subject variability, as indicated by high ICC values” |
| Stokes-Riner *et al*. 2007  Prospective; fertile population. | 615 | Main reason for variability: abstinence time  “…no a priori reason to believe the second sample would systematically differ from the first among infertile men when it did not among fertile men” |
| Mishail *et al*. 2009  Retrospective  Varicocele /abnormal | 112 | “the second SA remained consistently abnormal in 111 out of 112 (99.1%) patients”  “only one initial abnormal SA is sufficient for the evaluation and treatment consideration in the infertile man with varicocele” |
| Leushuis *et al.* 2010  Subfertile couples  Retrospective | 5240 | Conc. ICC: 0.89; motility ICC 0.59; morphology ICC 0.60 ; volume 0.70. High reliability for conc, moderate for motility and morphology percentage with discordant results ranged from 6% to 21% |
| Christman *et al*. 2013  Retrospective  Young patients; risk group:  Varicocele / cryptorchidism | 79 | CVw from 36% (volume) to 82% (Total Motility Count, TMC)  ICC from 0.55 (% motile) to 0.88 (total number). ICC for TMC 0.78 [95% CI, 0.67 – 0.85]: TMC ICC consistent with substantial reliability |

Conc:concentration; SA : Semen Analysis.

In three, otherwise high quality publications (sufficient detail in the Methods to determine appropriate quality control procedures), only the size of the variation is examined, not measures of correlation (e.g. ICC).

| **Reference** | **N** | **Conclusion** |
| --- | --- | --- |
| Keel 2006  Patients and donors  Multiple ejaculates | 139 | Only measures of variation, nothing on correlation |
| Amann and Chapman 2009  Retrospective  50 donors with ≥20 samples each | 50 | Presenting data (e.g. CV) as a base for calculations on how many ejaculates that need to be assessed to obtain a reliable (exact) number, presumably related to sperm production and sperm output  At least three samples from a hypothetical future subject are recommended |
| Jarow, Fang, and Hammad 2013  Pooled data, placebo RCT | 333 | Mean CVw for semen parameters 10% to 50%. Only variability, no data on correlation and repeatability.  The reduction in variability with an increasing number of samples per time point had decreasing returns beyond two samples.  “… considerable variation in semen parameters with time in subjects who received placebo.” |

A recent study by Zhu and colleagues (Zhu *et al.,* 2016) specifically addressed one versus two semen samples for epidemiology studies in 666 men. Although they concluded that there was no significant difference between first and second sample as far as can be judged by pairwise comparisons for concentration, total number, rapid progressive, all progressive and normal morphology, there was insufficient detail presented in the Methods to determine the quality control procedures performed in the ejaculate analysis. As such this paper was not included.

*Summary and Conclusion*

Investigations that have included measures such as ICC suggest that analysis of a single ejaculate is sufficient to determine the most appropriate investigation and treatment pathway. However, if the aim is to establish a “true” value of a more exact sperm production such as sperm output rate, then a single ejaculate is insufficient (see Amann and Chapman et al 2009).

*References*

Amann RP and Chapman PL. Total sperm per ejaculate of men: obtaining a meaningful value or a mean value with appropriate precision. *J Androl* 2009: **30**; 642-649.

Christman MS, Kraft KH, Tasian GE, Zderic SA, and Kolon TF. Reproducibility and reliability of semen analysis in youths at risk for infertility. *J Urol* 2013: **190**; 683-688.

Francavilla F, Barbonetti A, Necozione S, Santucci R, Cordeschi G, Macerola B, and Francavilla S. Within-subject variation of seminal parameters in men with infertile marriages. *Int J Androl* 2007: **30**; 174-181.

Jarow JP, Fang X, and Hammad TA. Variability of semen parameters with time in placebo treated men. *J Urol* 2013: **189**; 1825-1829.

Keel BA. Within- and between-subject variation in semen parameters in infertile men and normal semen donors. *Fertil Steril* 2006: **85**; 128-134.

Kim SK, Lee JM, Jee BC, Suh CS, and Kim SH. Semen quality of consecutive ejaculates from cancer patients for fertility preservation. *Fertil Steril* 2014: **102**; 1124-1129 e1123.

Leushuis E, van der Steeg JW, Steures P, Repping S, Bossuyt PM, Blankenstein MA, Mol BW, van der Veen F, and Hompes PG. Reproducibility and reliability of repeated semen analyses in male partners of subfertile couples. *Fertil Steril* 2010. **94**:2631-2635.

Mishail A, Marshall S, Schulsinger D, and Sheynkin Y. Impact of a second semen analysis on a treatment decision making in the infertile man with varicocele. *Fertil Steril* 2009: **91;** 1809-1811.

Stokes-Riner A, Thurston SW, Brazil C, Guzick D, Liu F, Overstreet JW, Wang C, Sparks A, Redmon JB, and Swan SH. One semen sample or 2? Insights from a study of fertile men. *J Androl* 2007: **28**; 638-643.
